# New pharmacotherapy options for noninfectious posterior uveitis

**DOI:** 10.1007/s10792-021-01763-8

**Published:** 2021-02-25

**Authors:** Uwe Pleyer, Piergiorgio Neri, Christoph Deuter

**Affiliations:** 1 Department of Ophthalmology, Charité – Universitätsmedizin, Berlin Institute of Health, 13353 Berlin, Germany; 2 Cleveland Clinic Abu Dhabi, Abu Dhabi, UAE; 3 Cleveland Clinic Lerner College of Medicine of Case Western Reserve University, Cleveland, OH USA; 4 Centre for Ophthalmology, University Hospital, 72076 Tuebingen, Germany

**Keywords:** bDMARD, Corticosteroid, nbDMARD, tsDMARD, Therapy, Uveitis

## Abstract

**Introduction:**

Noninfectious inflammation of the posterior eye segment represents an important cause of visual impairment. It often affects relatively young people and causes a significant personal and social impact. Although steroids and nonbiologic-

Disease-Modifying Antirheumatic Drugs (nbDMARDs) are effective both in acute and long- lasting diseases, however they are increasingly being replaced by biologic (DMARDs). bDMARD. This article therefore aims to identify recent advances in the therapy of noninfectious posterior segment uveitis.

**Methods:**

A Medline-search was conducted using the terms: nbDMARD, bDMARD, posterior uveitis, intermediate uveitis, treatment, corticosteroid. In addition, clinical studies were included as registered at ClinicalTrials.gov.

**Results:**

Currently two major lines of treatments can be identified: (1) the intraocular application of anti-inflammatory agents and (2) the introduction of new agents, e.g., (bDMARDs) and small-molecule-inhibitors. Whereas intravitreal treatments have the advantage to avoid systemic side effects, new systemic agents are progressively earning credit on the basis of their therapeutic effects.

**Conclusion:**

Even when current treatment strategies are still hampered by the limited number of randomized controlled trials, promising progress and continuous efforts are seen.

## Introduction

Although uveitis is considered an orphan disease, around 290 Mio people worldwide are visually impaired, and 40 Mio are severely affected and blind because of intraocular inflammation [1] (www.who.int/blindness). The proportion of young people affected by uveitis is much higher than elderly ones, representing the fourth leading cause of blindness in the working age. As a consequence, health care systems are significantly affected due to the high cost of medications, medical and social care. By definition, uveitis is a broad term of inflammation of the uveal tract, which also may affect other intraocular structures including retina, retinal vessels, vitreous body, and optic disk. The Standardized Uveitis Nomenclature (SUN) classified uveitis on a morphological basis, focusing on iris/ciliary body, vitreous, and retina/choroid involvement [2]. The etiological classification differentiates uveitis into 2 different types: infectious and non-infectious.

Although the pathogenesis of noninfectious uveitis is still not completely understood, it is agreed that it might be an autoimmune or immune-mediated response, leading to a chronic relapsing clinical course [3]. The hypothesis of an autoimmune pathogenesis is confirmed by experimental models. Retinal autoantigens may trigger an experimental autoimmune uveitis (EAU) which share similarities with uveitis in humans. The current core concept on uveitis pathogenesis is represented by the role played by Th CD4 + cells [4]. These activated T cells are essential pacemakers for the immune reaction, mediated by specific transcription factors and signature cytokines. In experimental models, both Th1 and TH17 lymphocytes play a central role in uveitis pathogenesis. More specifically, interferon (INF)-γ is synthesized by Th1 cells and activate non-specific mononuclear cells, leading to tissue infiltration and recruitment of neutrophil granulocytes. Interleukin-17 further mediates such immune reaction [5].

The heterogeneity of clinical manifestations of non-infectious uveitis is thought to be triggered by different antigens, where HLA-association may interplay in different ways with exogenous factors [6]. On the basis of clinical experience, a number of therapeutic approaches were considered. The top key considerations for the treatment of non-infectious posterior segment uveitis may be summarized as the following:

1. Rule out infectious agents as well as malignancies (masquerade syndrome)
2. Perform a correct anatomical classification
3. Consider laterality
4. Investigate a possible underlying systemic disease
5. Appropriately rank disease severity
6. Follow-up therapy both for safety and efficacy

A stepladder approach is commonly followed in order to control acute as well as relapsing noninfectious posterior uveitis. Although corticosteroids still remain the mainstay for uveitis treatment, their undisputed side effects in the long term are minimized by traditional steroid-sparing drugs. Calcineurin inhibitors, antimetabolites and cytotoxic agents may exert an effective long-term control of NIPU, even though often they may fail, and a further step has to be considered. Biologic agents (bDMARDs) opened a new era in ophthalmology and are widely used in many medical subspecialties including uveitis [7, 8].

Beside safety and cost/effectiveness issues, such drugs are prevalently used off label: very few of them were tested in randomized control trials (RCT) and this still represents their main limitation. However, the pharmaceutical industry shows interest in supporting and investing for the treatment of NIUP: local sustained drug delivery systems and novel systemic cytokines blockers are some examples on how the therapeutic scenario will change in the future.

This article is focused on chronic NIPU treatment, more specifically on intraocular anti-inflammatory/immune modulating agents as the following:

1. Intravitreal therapies
2. Systemic bDMARDs
3. Future treatments such as tsDMARDs

## Methods

This review is based on the findings of individual studies, meta-analyses, and Cochrane Reviews retrieved by a selective literature survey of the Medline and Google Scholar databases. The search was conducted using the terms: nbDMARD, bDMARD, posterior uveitis, intermediate uveitis, intravitreal, treatment, corticosteroid. In our search we included only articles published in English available until October 2020. In addition, clinical studies were included as registered at ClinicalTrials.gov.

## Results

In this article, 44 systematic reviews and 335 articles were evaluated that fulfilled the search strategies. In addition, we included 35 RCT that were registered and completed at ClinicalTrials.gov until October 2020. The overwhelming number of studies concerned intravitreal therapy (*n* = 206) while trials referring to bDMARDS (*n* = 109) or cDMARDs (*n* = 30) were found. The results of our research are structured according to: intravitreal therapy, conventional and biological DMARDs as well as an outlook on future therapy options. This includes tsDMARDS and new cell-based approaches.

## Intravitreal therapy

The intravitreal route allows to deliver the medication closer to the affected site in posterior, intermediate and panuveitis. In addition, intravitreal therapy might be considered in cases where contraindication to systemic drugs or comorbidities may create issues. While bilateral chronic uveitis is often treated by systemic approach, particularly when associated with a systemic disease (e.g., sarcoidosis), unilateral non-infectious uveitis is firstly treated by intravitreal therapy [9].

Although intravitreal drugs are developed as possible first-line therapy, they are often used as adjuvant agents to systemic immunomodulation, such as in case of either sight-threatening complications like cystoid macula edema (CME) or sub-optimal response. However, the main flaw of intravitreal therapy is represented by its limited duration.

Although many different molecules are under investigation (overview in Table 1), corticosteroids are still the only ones currently available. They are either injected as inserts by a 22-or 25-gage injection applicator, or surgically implanted as drug delivery system for a sustained release.

**Table 1:** Intravitreally administered compounds in clinical use or in development for posterior segment uveitis

Drug class/compound	Mechanism of action	Status of development	Manufacturer/developer
*Corticosteroid*			
0.59 mg fluocinolone acetonide implant (Retisert®)	Decreased transcription of proinflammatory cytokines and chemokines, reduction of increased capillary permeability, inhibited migration of immune cells, etc.	Approved (US only)	Bausch + Lomb
0.7 mg dexamethasone implant (Ozurdex®)		Approved	Allergan
			Alimera
		Approved	
0.19 mg fluocinolone acetonide implant (Iluvien®)			
			Novartis
		Approved (US only)	
40 mg/ml triamcinolone acetonide suspension (Triesence®)			
*mTor inhibitor*			
Sirolimus (DE-109)	mTor/ reduces T and B cell activation and promotes regulatory T cell differentiation	Phase III ongoing, resubmitted (NCT03711929)	Santen
*DHODH inhibitor*			
PP-001	Inhibition of DHODH reduces activated T-cell proliferation and inflammatory cytokine release	Phase I (EudraCT-No. 2016–000,412-15)	Panoptes pharma
*Antimetabolite*			
Methotrexate	Inhibits dihydrofolate reductase; both B and T cell suppression	Case series (off-label for intravitreal use) (Ref…….)	Medac, Pfizer
*TNF-alpha inhibitors*			
Infliximab	Chimeric monoclonal IgG antibody against TNF-alpha	Case series (off-label for intravitreal use) (Ref…….)	MSD
Adalimumab	Human monoclonal IgG antibody against TNF-alpha	Case series (off-label for intravitreal use) (Ref…….)	AbbVie
*VEGF inhibitors*			
Ranibizumab (Lucentis®)	Monoclonal IgG antibody fragment against VEGF-A	Case series (off-label for uveitis, except choroidal neovascular membrane secondary to uveitis)	Genentech/Novartis
Aflibercept (Eylea®)	Recombinant fusion protein against VEGF-A and VEGF-B	Case series (off-label for uveitis)	Regeneron/Bayer
Bevacizumab (Avastin®)	Monoclonal IgG antibody against VEGF-A	Case series (off-label for intravitreal use) (Ref…….)	Genentech

*DHODH* Dihydroorotate dehydrogenase; *TNF* Tumor necrosis factor; *VEGF* Vascular endothelial growth factor

Although their efficacy is undisputed, steroid implants are carefully used under the light of their potential local side effects: elevated intraocular pressure (IOP) and possible development of lens opacity, particularly for younger patients, are relatively common.

The first intravitreal drug delivery system for uveitis was developed almost twenty years ago. It is a 0.59 mg fluocinolone acetonide implant (Retisert®) with a sustained drug release over 30 months approximatively. The device is implanted via pars plana incision and fixed with sutures to the sclera, similarly to ganciclovir implant (Vitrasert®) used for the cytomegalovirus retinitis in the past. In a controlled phase II/III clinical trial, Retisert® demonstrated superior control of inflammation compared to conventional immunosuppressives [10]. However, within 3 years after implantation nearly all phakic patients required cataract surgery and 37% underwent glaucoma surgery. Retisert® is currently approved in the USA only.

Recently, two further steroid drug delivery systems were added to the therapeutic armamentarium. Ozurdex® is a biodegradable 0.7 mg dexamethasone insert approved for the treatment of non-infectious posterior segment uveitis both in USA and in Europe. Albeit the initial phase II/III study claimed a duration of action of up to 26 weeks, clinical experience has shown a need of re-injection within 3–4 months [11]. Although approved for the treatment of the underlying intraocular inflammation, a survey among uveitis specialists identified uveitic CME as the preferred indication [12, 13]. Ozurdex® [14] appears less prone to high intraocular pressure and cataract occurrence than Retisert®. Iluvien® is a non-bio-degradable, 0.19 mg fluocinolone acetonide implant lasting up to 36 months [15].

Both Ozurdex® and Iluvien® should not be used in aphakic eyes, since an anterior chamber dislocation of the implants may occur, threatening corneal endothelium. In such cases, 1–4 mg of a triamcinolone acetonide suspension (Triesence®) can be considered [16].

Now a days, clinical trials on intravitreal immunosuppressive drugs are on-going. A current candidate is the mTor inhibitor sirolimus [17, 18]. Sirolimus is a well-known immune modulatory agent targeting T-cell differentiation (Powel). A phase III trial [SAKURA] was conducted assessing the safety and efficacy of intravitreal sirolimus for treatment of active, noninfectious posterior uveitis [19]. First applications for approval to EMA and FDA were withdrawn or rejected in 2016 and 2017. However, in 2018 the LUMINA Phase III study (NCT03711929) was introduced to extend previous experience and this multicenter trial is expected to be completed in 2022.

Currently, the safety of PP-001, a small molecule which inhibits dihydroorotate dehydrogenase (DHODH) is under investigation. Based on preclinical data [20] in an EAU model, the agent has undergone a multicentric trial in Europe but no results are available so far.

The intravitreal use of nbDMARDs and bDMARDs, such as methotrexate, infliximab and adalimumab, is just limited to anecdotal reports in the medical literature [21–24]. A warning in using them routinely has to be made due to the lack of consistent evidence of both their safety and efficacy.

The vascular endothelial growth (VEGF) inhibitors ranibizumab (Lucentis®) and aflibercept (Eylea®) were developed and approved for intravitreal use for wet age-related macular degeneration, diabetic macular edema and macular edema secondary to retinal vein occlusion. Intravitreal administration of bevacizumab (Avastin®) for the same indications was used off-label before the previous two were approved. A series of small case series showed promising results also in uveitic macular edema, even though repeated injections were often needed [25–31].

The advantage of local therapies in noninfectious uveitis is undisputed. There is an unmet need for the use of non-steroidal immunomodulatory drugs for intravitreal application, even though short-term efficacy might remain an issue for a while.

## Nonbiologic DMARDs

DMARDs can be classified into nonbiologic (nbDMARDs) and bDMARDs. The nbDMARDs most commonly used in uveitis patients are listed in Table 2. Based on their pharmacological properties, they can be subdivided into antimetabolites (methotrexate, azathioprine and mycophenolate mofetil), calcineurin inhibitors (ciclosporine A and tacrolimus) and alkylating (cyclophosphamide and chlorambucil). Since nbDMARDs reach the optimal level of activation slowly, they have to be combined with systemic steroids at baseline. Based on the Systemic **I**mmunosuppressive **T**herapy for **E**ye Disease (SITE) cohort results inflammation control can be achieved in 52–76% of patients taking nbDMARDS within one year [32]. Systemic steroid dose could be reduced to 10 mg/day or less in the majority of patients [33–37]. Since nbDMARDs are well established and have been frequently reviewed in detail and in the interest of limited space, we refer to previous reviews [38, 39] see Table 2 for an overview of the drugs discussed in this section.

**Table 2:** Non-biologic DMARDS for ocular inflammation [mod. from *]

Class	Generic name	Dosage	Drug administration	Onset of action	Mechanism	Common side effects and
Anti-metabolites	Azathioprine	1–3 mg/kg/day	Oral	1–3 months	Changes purine metabolism Strong T cell and weak B cell suppression	Bone marrow suppression, gastrointestinal upset, hepatotoxicity,
	Methotrexate	10–25 mg/week	Oral, subcutaneous or intramuscular injections	2 weeks to 3 months	İnhibits dihydrofolate reductase Both B and T cell suppression	Bone marrow suppression, gastrointestinal upset, hepatotoxicity, alopecia
	Mycophenolate mofetil	500–1000 mg BID	Oral	2 weeks to 3 months	Inosine-5′monophosphate inhibitor B and T cell suppression	Diarrhea, nausea, neutropenia, infection
Calcineurine inhibitors	Cyclosporine A	2–5 mg/kg/day	Oral	2–6 weeks	Inhibits calcineurin and signaling pathways T cell mediated immune response	Neprotoxicity,hypertension,tremor,gum hyperplasia, hirsutism
	Tacrolimus	0.15 mg/kg BID increasing to 2–3 mgm BID	Oral	2–6 weeks	Inhibits calcineurin and signaling pathways T cell mediated immune response	Neprotoxicity,hypertension,tremor,gum, neurotoxicity,hepatitis,hyperkalemia, diabetes
Alyklating agents	Cyclophosphamide	1–2 mg/kg/day	Oral intavenous	2–8 weeks	Cross links DNA, block DNA replication and cell division B and T cell suppression	Bone marrow suppression, hemorrhagic cystits,sterility, alopecia, increased risk of malignancy
	Chlorombucil	0.1 mg/kg/day	Oral	4–12 weeks	Cross links DNA, block DNA replication and cell division Particulary B cell division	Bone marrow suppression,sterility, increased risk of malignancy

*Pleyer U, Pohlmann D, Kardeş E, Poddubnyy D, Rademacher J

Emerging drugs for the treatment of noninfectious uveitis

Expert Opin Emerg Drugs. 2019; 24:173–190

## Biologic DMARDs

The progresses on the knowledge of uveitis pathogenesis have brought novel molecular targets for the modulation of ocular immune response. In the early 1990s, bio-molecular engineering came up with new immunosuppressive drugs called biologics (bDMARDS), representing a revolution in the treatment of several medical areas. Those molecules had the specific property to bind pro-inflammatory cytokines (Table 3), leading to a fast and effective control of inflammation.

**Table 3:** Biologic DMARDS for ocular inflammation [ modified from reference 7]

Name *(commercial name)*	Indications	Type of protein	Target	Ocular diseases or condition	Recommendation Level
Etanercept (Enbrel)*	RA, PSA, Psoriasis, JIA	Fusion Protein	TNF-α	NIU, sarcoidosis	B
Golimumab *(Simponi)*	RA, AS, PSA, Ulcerative Colitis	Monoclonal Antibody	TNF-α	NIU	C
Adalimumab *(Humira)*	NIU, RA, AS, PSA, Psoriasis, CD	Monoclonal Antibody	TNF-α	NIU (including different uveitis entities: BD, idiopathic uveitis, sarcoidosis, BSRC, TINU syndrome, VKH disease, pars planitis; other: HLA-B27, JIA)	A
Infliximab *(Remicade)*	RA, AS, PSA, Psoriasis, CD	Monoclonal Antibody	TNF-α	BD Pediatric NIU (uveitis entities include JIA, BD, sarcoidosis, VKH disease) Other uveitis entities (including BD, BSRC, sarcoidosis, idiopathic vasculitis, VKH disease)	B
Certolizumab-pegol *(Cimzia)*	CD, RA, PSA, AS	Monoclonal Antibody	TNF-α	NIU	C
Anakinra *(Kineret)*	RA	Recombinant, modified Human Interleukin-1 Receptor Antagonist by one methionine added to its N-terminus	Interleukin-1 receptor	BD	C
Canakinumab (Ilaris)	CAPS, JIA	Monoclonal Antibody	Interleukin-IL-1β	BD	C
Gevokizumab (Xoma)	None at the moment	Monoclonal Antibody	Interleukin-IL-1β	NIU	B**
Daclizumab *(Zenapax)*	Prevention of renal transplant rejection	Monoclonal Antibody	Interleukin-2 receptor	NIU (including different uveitis entities such as: idiopathic anterior uveitis and panuveitis; MCP; scleritis, idiopathic panuveitis; sarcoid; panuveitis; HSV-associated anterior scleritis; idiopathic kerato- uveitis)	B
Tocilizumab *(Actemra)*	RA, JIA	Monoclonal Antibody	Interleukin-6 receptor	NIU (including different uveitis entities) JIA associated uveitis	B
Sarilumab (Kevzara)	RA	Monoclonal Antibody	Interleukin-6 receptor	NIU (including different uveitis entities)	B
Secukinumab *(Cosentyx)*	RA, Psoriasis	Monoclonal Antibody	Interleukin-17A	NIU (including different uveitis entities: BD uveitis noninfectious; non-BD uveitis; quiescent, noninfectious, non-BD uveitis)	B**
Abatacept *(Orencia)*	RA	immunoglobulin CTLA-4 Fusion Protein	CD80, CD86	NIU	C
Rituximab *(Rituxan)*	CD20-positive NHL, B-CLL, RA	Monoclonal Antibody	CD20	BD, NIU, scleritis	C
Alemtuzumab *(Campath-1H)*	B-CLL	Monoclonal Antibody	CD52	NIU, BD	C
Interferons (Betaferon, Intron A, Pegintron, Roferon)	MS, Melanoma, Hepatitis C	Signaling protein	Multiple biologic targets	NIU, CMO, BD	C

*AS* Ankylosing spondylitis, *BD* Behçet disease, *BCLL* B-cell chronic lymphocytic leukemia, *BSRC* Birdshot retinochoroiditis, *CAPS* Cryopyrin-associated periodic syndromes, *CD* Crohn’s disease, *JIA* Juvenile idiopathic arthritis, *MCP* Mucous Cicatricial Pemphigoid, *MS* Multiple Sclerosis, *NHL* Non-Hodgkin’s lymphoma, *NIU* Noninfectious uveitis, *PSA* Psoriatic arthritis, *RA* Rheumatoid arthritis, *TINU* Tubulo-Interstitial Nephritis and Uveitis *VKH* Vogt-Koyanagi-Harada

*Discontinued use for uveitis since suspected in triggering de novo uveitis

**Failed registration trial

Adapted from [7]

Tumor Necrosis Factor (TNF)-α plays a central role in the pathophysiology for many diseases, such as rheumatoid arthritis (RA), juvenile idiopathic arthritis (JIA), inflammatory bowel disease (IBD) and non-infectious uveitis (NIPU) [40]. TNF-α is primarily synthesized by activated macrophages, T lymphocytes, and natural killer (NK) cells [41]. TNF-α specifically binds either TNF-receptor 1 (TNF-R1) or TNF-receptor 2 (TNF-R2) and up-regulates the expression of endothelial adhesion molecules [42]. Uveitis presents high levels of TNF-α in intraocular fluids which is directly proportional to CD4 + Th cells activation [43], leading to potentially irreversible tissue damage.

Albeit many different bDMARDs targets have been explored, anti-TNF-α agents are still considered the most effective therapeutic weapons in immune mediated ocular disorders. At this time, the armamentarium of systemic anti TNF-α agents is represented by first generation drugs such as etanercept, infliximab, adalimumab, and the second-generation ones golimumab and certolizumab. Etanercept is a DNA recombinant fusion protein which combines TNF receptor to the constant end of the IgG1 antibody and binds the TNF-α-R2 which was immediately abandoned for uveitis treatment, since it seemed to trigger de novo uveitis in patient affected by rheumatological diseases, even though this occurrence was recently put again under discussion [44].

The first TNF-α agent which raised the attention of researchers was Infliximab which is a mouse-human chimeric antibody targeting TNF-α. Several studies reported remarkable benefits in treating severe and resistant intra-ocular inflammation both in adults and pediatric patients [45, 46]. The typical loading dose ranges in between 3 and 5 mg/kg body weight intravenously, up to 10–20 mg/kg. A high number of publications have proven Infliximab as a safe and effective immunosuppressive agent for patients with refractory uveitis [47]. However, even though the infusion interval should follow a specific regimen, infliximab was proven safe and effective at higher doses even, by shortening intervals or by increasing the dose per infusion in case of inadequate response [48]. In addition, it is common practice to administer concurrent antimetabolites with infliximab to decrease anti-chimeric antibody formation and increase the duration of drug efficacy as well [49]. However, infliximab can be used exclusively off label in uveitis patients.

Currently, the only approved TNF-blocker is Adalimumab. Adalimumab is a fully human anti TNF-α monoclonal antibody blocking TNF-α and TNF R1 and TNF R2 interaction, generated by phage display technology [50]. Adalimumab was the very first TNF-α monoclonal antibody which is given via subcutaneous injection. Such new approach was acknowledged to be revolutionary for a more user-friendly administration [51].

Adalimumab dose is 80 mg at base line and then 40 mg every other week given at home, with no need of admission. The subcutaneous route lowers the risk of a possible sudden allergic reaction and optimizes patient’s quality of life. Similarly to other anti-TNF-α agents, Adalimumab previously was used off label for many inflammatory diseases.

However, differently than other anti-TNF-α drugs, Adalimumab has been tested in three large randomized control trials for uveitis. All of them showed Adalimumab as an effective drug in controlling both active (VISUAL I) [51] and inactive (VISUAL II) [52] uveitis. Moreover, treatment extension trial (VISUAL III) [53] represented the real-life model which put Humira as the frontline bDMARDs agent approved for non-infectious intermediate, posterior and panuveitis as up-to-date. As it was described for Infliximab, recent reports provide evidence of a favorable response to Adalimumab dose escalation for patients sub optimally responding to standard regimen [54, 55].

All the other anti-TNF-α monoclonals such as Golimumab and Fab´-fragment conjugated with polyethylene glycol agents, such as Certolizumab-pegol, have been reported to have a certain effect on uveitis, albeit the power of those studies limits the judgement of such reports as anecdotal at the moment.

At the same time, a series of reports on other bDMARDs targets, such as interleukin (IL)-2 and IL-1β, have been published, under the light of a certain role played by such interleukins in experimental models of autoimmune uveitis. Data extrapolated from trials on anti-IL1RA use for different autoimmune syndromes have shown a beneficial effect, as well as a promising profile for both tolerability and safety [56]. In particular, anti-ILs safety profile for tuberculosis-related serious adverse events seems to be much better compared to TNF-α inhibitors. This advantage significantly increased the interest of researchers on such bDMARDs agents [57]. Anakinra is a specific receptor antagonist of IL-1, given subcutaneously at a dose of 100 mg per day. The efficacy of Anakinra for auto-immune uveitis has been shown in animal model [57], although clinical experience is limited to case reports and it might be indicated in autoinflammatory diseases only, like chronic infantile neurological cutaneous articular syndrome (CINCA)-associated uveitis unresponsive to anti-TNF therapy [58].

Canakinumab is a fully human anti-IL-1β-antibody which has indication for systemic JIA and cryopyrin-associated periodic syndromes (CAPS). Beside a series of anecdotal case reports, the drug has been reported as effective in series of publications mainly by a single group of researchers from Italy, albeit those reports present a poor evidence by extrapolating the data and the cohorts of patients treated are very heterogeneous [59]. This evidence should suggest prudency in using the drug tout court, considering also the fact of a very unfavorable cost/effectiveness profile.

Gevokizumab is a humanized monoclonal antibody targeting IL-1β. Although a pilot study on active Behçet Disease (BD)-related uveitis and retinal vasculitis non-responder to traditional immunosuppressants has shown a rapid and sustained reduction of intraocular inflammation [60], gevokizumab sadly failed to meet the primary outcome measures in phase III multicenter clinical trials for inactive noninfectious uveitis (EYEGUARDTM-C), active noninfectious uveitis (EYEGUARDTM-A), and BD associated uveitis (EYEGUARDTM-B) [61].

Daclizumab is a monoclonal antibody, binding a heterotrimeric protein called IL-2 receptor, expressed on cellular surface of natural killer cells and T- and B-lymphocytes. Despite initial promising results on uveitis treatment [62], Daclizumab is no more available on the market due to both lack of demand and to possible risk of encephalitis.

More and more interest is now ramping up for anti-IL-6 agents. Innate **cells,** such as monocytes and macrophages, are the main producers of IL-6, even though **T cells** may generate IL-6 during chronic inflammation [63]. IL-6 which plays multiple functions such as modulator of T-cell activation and Immunoglobulins secretion, as well as leukocyte recruitment. [64].

Tocilizumab targets specifically IL-6 Receptor and was mainly used intravenously for RA at the dose range of 4–12 mg/kg every 2–4 weeks, even though its subcutaneous route was recently approved. The efficacy of Tocilizumab in controlling Behcet disease -related uveitis has been shown as well [65]. Recently Tocilizumab has been proven to be particularly effective in the treatment of CME and was successfully used in patients who were previously refractory to both nbDMARDs and anti-TNF agents. [66].

Sarilumab, a novel recombinant humanized *anti*-*IL-6* receptor monoclonal antibody, was recently introduced for the control of NIPU in a phase II trial (SARIL-NIU). The trial’s goal aimed to assess the efficacy and safety of subcutaneous Sarilumab 200 mg, given every other week for the control of NIPU. Sarilumab showed similar qualities compared to Tocilizumab by providing clinical evidences of good control of posterior segment uveitis, as well as of CME [67]. However, more evidence is needed in larger trials.

Rituximab is a mouse-human chimeric monoclonal IgG1 antibody targeting B-lymphocyte antigen CD-20, which achieved the indication for the treatment of relapsing or refractory non-Hodgkin lymphoma [68] by Food and Drug Administration (FDA) in 1997. Although Rituximab acts toward B-cells, it exerts an excellent control of T cell-mediated autoimmune diseases [69]. Despite the interesting profile, Rituximab presents only a low level of evidence for its efficacy since the largest cohort treated is composed by 11 patients so far [70].

Other molecular targets were explored in the past years. Unfortunately, some drugs such as Alemtuzumab targeting CD52 [71] and Abatacept a soluble fusion protein targeting cytotoxic T-lymphocyte-associated antigen (CTLA)-4 [72] did not find space due to either lack of efficacy or safety profile. Great expectancies were given to interfere with the IL-17 pathway. Secukinumab is a human IgG1 monoclonal antibody which selectively binds to the interleukin-17A (IL-17A) cytokine and inhibits its interaction with the IL-17 receptor. Unfortunately, it failed to meet the primary outcome measures and the clinical trials for uveitis were discontinued. Notablely, the trial was conducted by administering the drug sub-cutanously, while a more recent report on an intravenous route has shown promising results and might lead to further investigations [73].

Interferon (IFN) covers a family of cytokines which are successfully used for severe uveitis. It is interesting to see on how TNF-α and IFN-α interplay by mutually cross regulating: TNF-α downregulates plasmacytoid dendritic cells activation and, as a consequence, IFN-α expression [74].

More specifically, INFs bind to INF receptors of different cells types, including not only immune system cells, but also cells of central nervous system, liver and lung among the others [75]. The route of administration is subcutaneous ranging from 3 to 6 million IU/day, at a frequency between three times weekly and once daily, even though there are some reports where the dose was ramped up to 9 million IU/day. IFN-α and -β were used in several ocular inflammatory diseases since long time. IFN-α therapies appear to have a profile close to anti-TNF-α bDMARDs agents, in terms of fast acting and controlling ocular inflammation, such as in ocular Behcet disease and refractory CME [76, 77]. Both IFN-α2a and -β1a were proven effective in resolving uveitic CME, even though most of the trials were dedicated in studying IFN- α2a. Moreover, IFN-α2a showed a promising long-term control of ocular inflammation, even after discontinuing the medication [78]. Unfortunately, IFN-α used in uveitis trials were recently discontinued due to low demand which was clearly a business-related decision, as per manufacturers statement. However, pegylated IFN-α2b was successfully used in a cohort of patient affected by severe uveitis in Behçet disease [79] and further investigations are recommended.

While IFN-β1a plays an established role for the treatment of Multiple sclerosis (MS) and MS-related optic neuritis, it showed to be effective in controlling both intraocular inflammation and CME [80], as well as recurrent punctate inner choroidopathy [81]. Recently the use of pegylated interferon has been proposed in small case series, but larger numbers are required in order to provide a scientifically correct advice [82].

### Limitations of bDMARDs and new developments

As with any other immune modulating approach, certain limitations apply for antibody-based agents. Not all patients show sufficient response and fulfill the expected treatment goals. “bDMARDs”, often are applied via the intravenous route and more importantly bear immunogenic potential that often results in loss of efficacy during continuous therapy by an anti-drug directed immune response. bDMARDs often have a long half-life that enhances their risk for adverse events as infections and malignancies. In addition, technologies used for their production are expensive and beyond the financial possibilities for all healthcare systems worldwide. Therefore, other options are explored, particularly for patients poorly or non-responsive to classical immunosuppressive agents or bDMARDs.

## Targeted synthetic DMARDs

Small-molecular-weight inhibitors have further enlarged the spectrum of treatment options and resulted in the introduction of a new term—targeted synthetic DMARDs (tsDMARDs). These substances are defined by a molecular weight of < 1 kDa and include several types of agents: phosphodiesterase inhibitors and kinase inhibitors. The term “JAK” was originally given as an acronym of "Just Another Kinase" Today the name is related to the Roman divinity Janus who has his two faces looking toward opposite directions. The principle of action of JAK-inhibitors is that they bind intracellularly to the cytoplasmic receptor domain of the type I and II cytokine receptors and then transmit the signal via the activation of signal converter and transcription activators (STATs). STATs consequently activate the transcription of target genes in the cell nucleus. This JAK/STAT pathway seems to be an important mechanism of immune mediated inflammatory pathways [83]. The four tyrosine kinases JAK1, JAK2, JAK3 and TYK2 form the family of JAKs. Up-to-date, several JAK inhibitors targeting different JAKs were introduced, among them the pan-JAK inhibitors Tofacitinib and Baricitinib, the selective JAK-1 inhibitor Upadacitinib and the specific JAK-1 inhibitor Filgotinib. Several alternative concepts are currently under investigation and have already shown promising preliminary results in other immune mediated diseases, including ocular disorders in RCTs [84–86].

Features of tsDMARDs differ in several aspects from monoclonal antibodies (mAbs) which may favor their future use.

- Where as bDMARDs are large proteins (approx. 150,000 Da) and are applied subcutaneously or intravenously, tsDMARDs are orally available.
- As a major further advantage of their smaller molecular weight (around 500 Da) they can enter easily cell walls. In addition, they may allow intraocular access via topical route.
- This also applies to intraocular access and crossing of the blood-retinal barrier, differently than bDMARDs, which present a significantly higher molecular weight. As a result, the therapeutic effect of mAbs in uveitis is probably limited to peripheral targets, while tsDMARDs can have a broader effect, specifically in the site of disease occurrence.
- In contrast to bDMARDs, tsDMARDs are not considered immunogenic. Immunogenicity represents the main issue of bDMARDs long-term use, leading to faster drug clearance and earlier loss of effectiveness, even for humanized ones.
- Another important advantage of tsDMARDs at a global perspective is the affordable cost for healthcare systems. Moreover, the development of bDMADRs is complex and obviously much more expensive than tsDMARDs production, which is the core discussion for any healthcare worldwide.

In the meantime, a broad spectrum of tsDMARDs have been investigated and approved as effective in the treatment of a series of diseases, such as hematological disorders and RA. Since the list of immune modulating tsDMARDs s has grown extensively, we are confident for certain on some interesting results in the near future.

### Targeting Janus kinases

JAK kinases interfere with proinflammatory cytokines (Table 4), rising the interest of researchers on their efficacy in a variety of immunological diseases.

**Table 4:** Small molecule compounds in development or clinical use for treatment of uveitis

Drug class/compound	Molecular target/ mechanism of action	Status of development (Reference)	Route of application	Originator /developer
Alpha 4 integrin inhibitors α4-api	Alpha 4 integrin/reduces T lymphocytes recruitment and inflammatory cytokine production	Preclinical [95]	Intraperitoneal	Cytel
Aldose reductase inhibitor BF5m	Aldose reductase/reduces oxidative stress and NF-κB signaling-related inflammatory cascade	Preclinical [96]	Intravitreal	University of Pisa, Italy
STAT3 inhibitors ORLL NIH001	STAT3/ suppresses Th17 differentiation and inflammatory cytokine production	Preclinical [97]	Intravenous	Orchid Research Laboratories Limited
AMPK analogs/activators AICAR	AMPK/ reduces NF-κB signaling, JAK-STAT signaling, leukocyte infiltration and cytokine synthesis	Preclinical [98]	Intraperitoneal	PeriCor Therapeutics, Schering-Plough
JAK inhibitors Tofacitinib (CP-690,550)	JAK-STAT cascade / reduces cytokine receptor signaling, immune cell survival, proliferation and function	Approved for RA by FDA and EMA; Phase III (NCT02592434) for JIA [99]	Systemic (PO)	Pfizer
Baricitinb (LY3009104)	JAK-STAT cascade/reduces cytokine receptor signaling, immune cell survival, proliferation and function	Approved for RA by EMA (NCT02265705)	Systemic (PO)	Elli lilly
Filgotinib	JAK-STAT cascade/reduces cytokine receptor signaling, immune cell survival, proliferation and function	Phase II clinical trial: active uveitis (NCT03207815)	Systemic (PO)	Gilead Sciences

*AMPK* Adenosine monophosphate activated protein kinase; *cAMP* Cyclic adenosine monophosphate; *DHODH* Dihydroorotate dehydrogenase; *JAK* Janus kinase; *JIA* Juvenile idiopathic arthritis; *mTor* Mammalian target of Rapamycin; *NF-κB* Nuclear factor κB; *PDE4* Phosphodiesterase 4; *PO* Per oral; *RA* Rheumatoid arthritis SIP, sphingosine-1 phosphate receptor; *STAT* Signal transducers and activators of transcription

Janus kinases (JAKs) family is not only involved in cell growth and differentiation in hematopoietic cell survival, but also affect immune cells such as lymphocytes.

The first two JAK inhibitors, Tofacitinib and Ruxolitinib, have been already approved not only for rheumatoid arthritis but are also for further inflammatory and immunological diseases, including psoriasis and severe dry eye [84–88]. Whereas classical bDMARDs target only one single cytokine, these tyrosine kinases inhibit intra-cellular signals from multiple cytokines. Currently available data show that various JAK inhibitors in patients with rheumatoid arthritis work comparably well in individuals who previously no longer responded to csDMARDs [89]. As a consequence of that, JAK inhibitors might even be able to play a primary role compared to conventional immune modulating agents or, at least, be more than a valid alternative in patients not responding to currently available therapies including bDMARDs. However, more reliable data from ongoing and future clinical trials are needed in order to better establish their therapeutic role in immunological disorders.

### Phosphodiesterase-4-inhibitors

Phosphodiesterase-4 (PDE4)-inhibitors belong also to tsDMARDs [90]. PDE4-inhibitors have immune modulating effects on cytokine expression during inflammation through downregulation of TNF alpha, IL-12 and IL-23. The prototype is Rolipram (ZK 62,711, Schering AG), which has been used as a precursor for the development of further agents. Rolipram targets a specific subtype of phosphodiesterase-4, leading to immunemodulation and anti-tumor effects. Initially, it has been proposed as a treatment for depression and multiple sclerosis but never approved for those diseases. Apremilast (Celegene Corporation) has been approved since 2014 for psoriatic arthritis and plaque psoriasis [91, 92]. An extension for the treatment of patients with oral ulcers associated with Behçet’s disease is currently pending. There are further promising data derived from treatment using Rolipram in EAU, but no data so far on clinical trials in uveitis [93, 94].

## Future therapies (gene therapy, cell-based therapy)

Gene therapy is an emerging therapeutic option for retinal disorders, including uveitis. Current clinical experience with monogenic retinal dystrophies is already available therefore opening new frontiers for other retinal diseases [100]. In experimental uveitis, successful interventions by gene therapy have long been proven. Therapeutic effects could be achieved in gene transfer to express interleukin-10, interleukin-1RA and TNF [101–103]. Current efforts to improve gene therapy focus on the identification of new vectors, novel therapeutic targets and the reliability of transfection. Significant efforts are still required in all areas. Previous applications have predominantly taken place with viral vectors, which are applied via a subretinal or intravitreal injection. Developments that pursue non-viral, non-invasive gene transfer are also of interest [103]. In this context, a clinical phase I/II study is worth mentioning, which is carrying out electro-transfection of the ciliary muscle, encoding the soluble human TNF-a p55 receptor in patients with posterior, intermediate and panuveitis (NCT03308045) [103].

However, dosage of the gene product, reliability of the transfection process and safety of prolonged transgene expression represent an unmet need so far [104].

A further interesting approach is the use of mesenchymal stem cells (MSCs). MSCs have a well-known immunomodulatory effects in autoimmune diseases by both their cell-to-cell interplay and ability to produce potent paracrine factors, such as cytokines, growth factors and exosomes. These effects play a key role in modulating regulatory T cells. EAU demonstrate the potential of MSCs in ocular inflammatory diseases by showing an obvious reduction both of severity and recurrence rate, even though conclusive data are not available yet [105–108]. A further evidence is the head to head comparison of a single MSC treatment proven as effective as repeated dexamethasone applications in the EAU model [109]. As a proof of the immunomodulatory effects, a significant reduction in T helper 1 (Th1) and Th17 was observed, while regulatory T cells were up-regulated.

## Conclusions

NIPU remains a challenge and is one of the major causes of preventable blindness. However, treatment of intraocular inflammation has never been so favorable and controllable as up to date. A number of conditions must already be critically evaluated at initial consultation. An accurate and early diagnosis, exclusion of infectious and masquerade syndromes is crucial. In addition, associated systemic diseases need to be considered initially and very likely will guide any therapeutic strategy.

Although steroids are usually necessary at baseline, they often lead to several complications particularly when used systemically. Following the approval of various intraocular steroid devices for intravitreal placement, the treatment of NIPU expanded significantly. More specifically, unilateral inflammation and CME are considered as indications for either dexamethasone or fluocinolone implant. As per systemic steroids, local steroids also may encounter some limitations due to the typical ocular side effects, such as secondary high IOP and cataract. A tailored decision making must therefore be taken, particularly in young patients who are more prone to be steroid responders. On the other hand, pseudo-phakic patients with no evidence of raised IOP after steroidal treatment are often good candidates to avoid systemic effects, particularly in elderly patients. The spectrum of intravitreal nonsteroidal agents may become more larger in the upcoming years. Some pioneering trails used a series of systemic immune-suppressive agents via intravitreal route and that opened a new way in interpreting their use.

Long-term management of intraocular inflammation is challenging with the unique scope of preserving vision and preventing recurrences. Patients either responding insufficiently to corticosteroids or developing adverse events, are commonly switched to nbDMARDs. According to international recommendations, steroid-sparing medications remain an essential component in the treatment strategy. Although this represents the common practice, there is no general agreement on which agent should be used, dosage ranging and treatment duration. Moreover, among the nbDMARDs only CsA is currently approved for uveitis.

The management of NIPU has certainly changed due to the worldwide approval of the TNF blocker adalimumab. In particular, TNF alpha seems to be still the most attractive therapeutic target, under the light of the broad efficacy of anti-TNF drugs across sub entities, including birdshot choroiditis, sympathetic ophthalmia and Behçet´s disease. Remarkable remissions occurred in patients with posterior uveitis failing all other treatments particularly e.g., Behçet´s disease with multisystem manifestations. Therefore, nonresponsive patients nowadays receive administration of bDMARDs.

However, the progressive increase in using TNF blockers revealed a series of issues. Although adverse events are relatively rare in uveitis patients, the risk–benefit ratio has to be carefully considered. Moreover, anti-drug neutralizing antibodies lead to lack of efficacy and this represent an important issue, as only one TNF agent is currently approved for NIPU. Switching to another anti-TNF creates an economic burden for certain: that is the main difference than other medical specialties which may count on a broader armamentarium of anti-TNF agents as per their label.

As a consequence of that, approval of a broader spectrum of bDMARDs, including other biologic targets, such as anti-IL-6, is now a days a priority. The scientific community looks forward to receiving the results of several phase II/III studies and, hopefully, their approval.

Those limitations might presumably be addressed by other agents, such as tsDMARDs. Promising results suggest JAK inhibitors as a valid alternative, as what happened in RA, where they seem to be equivalent to conventional immuno modulating agents and bDMARDs. Given their advantages as described above, such as inhibiting signal transduction for multiple cytokines simultaneously, oral availability and easier production, they may open a new therapeutic scenario. An outlook into the future should also be included in our assessment. First and foremost, therapeutic options which turn away from conventional pharmacological treatment should be mentioned. Gene therapy has been advocated already for many years. Currently, a variety of therapeutic applications through intravitreal or subretinal gene transfer have been approved already. This creates more than a hope for intraocular immunomodulatory approaches. In addition, cell-based modulation of the immune system may become an option. MSC seems to be an interesting approach, as per what is emerging by an ongoing phase I trial for immune modulation and prevention of corneal allograft rejection.

In summary, the current development of the treatment of intraocular inflammation offers fascinating perspectives, new hopes for many patients, which should also have a positive effect on their quality of life.
